# Copy number alteration signature defines undifferentiated pleomorphic sarcomas and leiomyosarcomas with poor prognosis

**DOI:** 10.1186/1753-6561-7-S2-P64

**Published:** 2013-04-04

**Authors:** Sara Martoreli Silveira, Rolando Rolando Andre Rios Villacis, Fabio Albuquerque Marchi, Mateus de Camargo Barros Filho, Sandra Drigo Linde, Cristovam Scapulatempo Neto, Isabela Werneck da Cunha, Ademar Lopes, Silvia Regina Rogatto

**Affiliations:** 1 Neogene Laboratory, CIPE, A. C. Camargo Hospital, São Paulo, Brazil; 2 Institute of Mathematics and Statistics, Inter-Institutional Program on Bioinformatics, São Paulo, Brazil; 3 Department of Pathology, Barretos Cancer Hospital (Pio XII Foundation), Barretos, Brazil; 4 Department of Anatomic Pathology, A. C. Camargo Hospital, São Paulo, Brazil; 5 Department of Pelvic Surgery, A.C. Camargo Cancer Hospital, São Paulo, Brazil; 6 Department of Urology, Faculty of Medicine, UNESP, Botucatu, São Paulo, Brazil

## Background

Undifferentiated high-grade pleomorphic sarcomas (UPS) display an aggressive clinical behavior with frequent development of distant metastasis and local recurrence. Since these tumors, particularly leiomyosarcomas (LMS), present a similar morphological pattern with other entities, the classification criterion by exclusion is frequently used.

## Methodology

In this study, array-based comparative genomic hybridization (array CGH) was applied in 20 UPS and 17 LMS (untreated cases). Array CGH (Agilent Technologies, 44K) data were analyzed by Nexus Software (v 6.0, BioDiscovery). Multivariate analysis was performed in order to identify the most important prognostic factors.

## Results

LMS presented lower frequency of genomic alterations in comparison with UPS. None of the variables were identified as independent prognostic factors, but gains at 1q21.3 were significantly associated with poor survival and showed almost significance as an independent prognostic factor (relative risk 13.8, *P* = 0.019). In addition, copy number profile of UPS and LMS was indistinguishable by unsupervised hierarchical clustering analysis, one out of three clusters presented cases associated with poor prognosis (*P* = 0.022). Relative copy number analysis for *ARNT*, *SLC27A3*, *PBXIP1* and *CCND1* genes was performed by quantitative real time PCR in 11 LMS and 16 UPS confirming the array CGH findings. Gains on 1q21-q22 involving *ARNT*, *PXIP1* and *SCL27A3* were observed exclusively in a subset of UPS.

## Conclusions

These findings describing a poor prognosis genomic signature in a subgroup of UPS and LMS can contribute to better stratify these patients for treatment.

## Financial support

FAPESP and CAPES.

